# An investigation of the therapeutic potential of the testicular tissue encapsulated in amnion membrane in mouse model: An experimental study

**DOI:** 10.18502/ijrm.v23i2.18489

**Published:** 2025-05-01

**Authors:** Keykavoos Gholami, Elahe Asheghmadine, Fateme Guitynavard, Leila Zareian Baghdadabad, Diana Taheri, Parisa Zahmatkesh, Leonardo Oliveira Reis, Seyed Mohammad Kazem Aghamir

**Affiliations:** ^1^Urology Research Center, Tehran University of Medical Sciences, Tehran, Iran.; ^2^Department of Plastic & Reconstructive Surgery, Cedars-Sinai Medical Center, Los Angeles, CA, USA.; ^3^Students' Scientific Research Center, Tehran University of Medical Sciences, Tehran, Iran.; ^4^Department of Pathology, Isfahan Kidney Disease Research Center, Isfahan University of Medical Sciences, Isfahan, Iran.; ^5^UroScience and Department of Surgery (Urology), School of Medical Sciences, University of Campinas, Unicamp, and Pontifical Catholic University of Campinas, PUC-Campinas, Campinas, S ao Paulo, Brazil.

**Keywords:** Amniotic membrane, Decellularized ECM, Hydrogel, Encapsulation, Testis.

## Abstract

**Background:**

Restoring fertility in male cancer individuals through testicular tissue transplantation faces challenges due to hypoxia-induced loss of spermatogonial stem cells (SSCs). Hydrogel encapsulation was explored to minimize hypoxic damage in testicular tissue transplantation. For this purpose, human amnion membrane (hAM)-derived hydrogel could be an alternative.

**Objective:**

The potential of hAM-derived hydrogel to support testis tissue grafts was evaluated.

**Materials and Methods:**

In this experimental study, testicular tissue samples (1–3 mm^3^) were obtained from 16 male NMRI mice (4–5 wk, 22 ± 2 gr). These tissue fragments were either encapsulated within a hydrogel derived from a hAM or left unencapsulated (control) prior to being autologously transplanted beneath the dorsal skin of mice subjected to hemilateral or bilateral orchiectomy. The grafted testicular tissues were histologically evaluated for key parameters, including the integrity of seminiferous tubules, survival of SSCs, Sertoli cell functionality, as well as hypoxia and apoptosis on day 21.

**Results:**

No significant differences were observed between groups regarding ST integrity, number of SSCs, Sertoli cell functionality, or the rate of hypoxia-inducible factor 1-alpha and apoptosis (p ≤ 0.05).

**Conclusion:**

In conclusion, this study demonstrated no effect of hAM hydrogel encapsulation on the outcomes of testicular tissue transplantation.

## 1. Introduction

Pediatric cancer treatments can permanently impact fertility. Cryopreserving immature testicular tissue (ITT), which contains spermatogonial stem cells (SSCs), is the only available fertility preservation method for prepubertal individuals (1). These cryopreserved tissues can then be used for future transplantation or in vitro maturation, as no sperm are produced before puberty (2). Fertility preservation through the freezing of ITT is now being practiced in centers of developed countries (3).

Ovarian tissue cryopreservation is an advanced clinical stage as a method for preserving fertility, while testicular tissue freezing remains in the experimental phase (4). For this purpose, preclinical studies have been conducted using fresh or frozen testicular tissue transplantation in animal models, which has paved the way for the clinical use of frozen ITT in the future (5). Recently, transplantation of frozen-thawed testicular tissue from prepubertal rhesus macaques enabled complete sperm production and offspring generation (6).

In this context, implanting frozen human testicular tissue into nude mice preserved the spermatogonia, allowing them to proliferate and differentiate (7, 8). However, studies, showed that testicular tissue transplantation in the form of auto or xenotransplantation faces important challenges since nearly 67% of SSCs are lost in the first 5 days after transplantation (9, 10). This can be due to the phylogenetic distance between species in the case of xenotransplantation, such as mice and humans, or the avascular grafting method because there is no surgical connection between the transplanted tissues and the vascular system of the host (11).

Neovascularization, linking transplanted capillaries with host vessels, is vital to prevent hypoxia, the main cause of early SSC reduction post-transplantation (12). Studies have investigated using hydrogels like alginate, chitosan, and fibrin to encapsulate testicular tissue, promote new blood vessel formation, and reduce hypoxia. These hydrogels often contain nanoparticles loaded with growth factors to stimulate angiogenesis (13, 14). This study investigates the use of hydrogel derived from decellularized tissues to improve the survival rate of autografted testicular tissues, an approach not previously reported. While testicular tissue hydrogels have been used to create 3D cultures and organoids, their use in human clinical studies is limited by ethical and legal constraints (15, 16).

Human amnion membrane (hAM) hydrogel is derived from the amniotic membrane and is rich in growth factors and extracellular matrix (ECM) proteins. Its anti-inflammatory and pro-angiogenic properties make it a promising biomaterial for tissue engineering, enhancing cell migration and vascularization in transplanted tissues. As a disposable obstetric tissue, hAM avoids ethical limitations and possesses various advantages, including the presence of ECM proteins, cytokines, growth factors, and anti-inflammatory properties (17).

In this study, testicular tissues were encapsulated in hAM hydrogel and autotransplanted to evaluate its potential for improving the efficiency of autologous transplantation of fresh testicular tissues in adult mice undergoing hemicastration (removal of one testicle) or bilateral castration (removal of both testicles).

## 2. Materials and Methods

### Study design

In this experimental study, which was conducted at the Urology Research Center of Tehran University of Medical Sciences, Tehran, Iran between 2022 and 2024, small fragments (1–3 mm^3^) of mouse testicular tissue from NMRI mice that had undergone hemilateral or bilateral castration (removal of one or both testes) were autotransplanted under the dorsal skin of the same mouse, with nonencapsulated grafts placed on the left and amnion membrane (AM) hydrogel-encapsulated grafts on the right. The grafts were then retrieved for analysis after 21 days.

### Sample size

According to the previous studies and considering the need to perform 3 biological replications and the possibility of losing mice during treatment, 8 mice in each group were selected for the study (14). Based on the confidence level of 95% and the standard deviation level of 1.7 derived from the above formula and based on the maximum error level of 0.9, the sample volume was estimated to be equal to 8 mice in each group.

Sample size = 2 (Z^α/2^ + Z^β^)^2^ × P (1-P)/(p_1_-p_2_)^2^

### Decellularization of AM

hAM were acquired from healthy newborns with their consent by approved protocols. The cryopreservation and decellularization of the AM were conducted in accordance with our established protocol (18, 19). In summary, the AMs were prepared by sectioning into small fragments and then frozen in a solution containing Dulbecco's Modified Eagle Medium (Gibco, USA), fetal bovine serum (Gibco, USA), and dimethylsulfoxide (Sigma, USA). Following thawing and washing for 15 min in 2x phosphate-buffered saline (PBS) (Sigma, USA), the tissue fragments underwent decellularization utilizing 0.01% sodium dodecyl sulfate (Sigma, USA) for 24 hr and then another 24-hr agitation in 1% Triton X-100 (Sigma, USA) solution while being intermittently rinsed. Subsequently, the tissue pieces were subjected to lyophilization for 24 hr before being turned into powder.

### Evaluation of decellularization protocol

Sections from decellularized tissue fragments were stained with hematoxylin and eosin (H&E) and then examined under a phase-contrast microscope. Hoechst staining and a fluorescent microscope were used to detect intact cell nuclei in decellularized and lyophilized AMs.

### Hydrogel formation

The ECM powder was combined with pepsin at a concentration of 1 mg/mL in 0.01 N hydrochloric acid (Sigma, USA), resulting in a 10 mg ECM/mL solution, and stirred at room temperature for 2 days. The pepsin- hydrochloric acid -ECM solution was then neutralized to pH 7.4 using 0.1 N sodium hydroxide (NaOH) (Sigma, USA) and made isotonic with a 10x PBS electrolyte solution. To assess gel formation in vitro, the neutralized AM pre-gel solution was incubated at 37 C for 1 hr (18).

### Tissue encapsulation and grafting

16 male NMRI mice, aged 4–5 wk, were included in the study. The animals were procured from the Pasteur Institute of Iran. Prior to the experiments, the animals were housed in a standard laboratory environment for 1 wk. The conditions included a temperature range of 22–25 C, a 12-hr light/dark cycle, and a relative humidity of 40–60%. The animals had unrestricted access to food and water during this acclimation period. The mice were anesthetized using an intraperitoneal injection containing ketamine (87.5 mg/kg) and xylazine (12.5 mg/kg) (Merck KGaA, Germany). Bilateral castration or hemicastration was performed along with the creation of skin flaps on their back skin during the same surgery. Tissue encapsulation and grafting were carried out in accordance with a previous study (13). Each side received transplantation of one fragment (1–3 mm³) either encapsulated in nonhydrogel or AM hydrogel, following incubation at 37 C/5% CO_2_ for approximately 25 min after being placed in physiologically balanced AM hydrogel (6 mg/mL). After 21 days, euthanasia by cervical dislocation was carried out, and grafts were harvested and fixed directly in a solution consisting of up to 4% paraformaldehyde, followed by analysis.

### Seminiferous tubule integrity

Seminiferous tubule integrity was evaluated by examining H&E-stained sections under a light microscope at a × 400 magnification. All seminiferous tubules (STs) were examined and categorized based on their condition. Intact tubules exhibited: i) cells adhering to the basement membrane, ii) cohesive cell structure, and iii) absence of sclerosis (score 1). Satisfactory tubules allowed for individualized intratubular cells despite focal necrosis being present (score 2). Damaged tubules displayed complete necrosis (score 3).

### Histological and immunohistochemical analysis

After fixation in 4% paraformaldehyde, the samples were preserved in paraffin and sliced into sequential sections of 5 μ m thickness. These sections were then placed on superfrost plus slides for immunohistochemistry analysis. Deparaffinization and rehydration of tissue sections were followed by a 30-min incubation at RT with a mixture containing 10% normal goat serum and 1% bovine serum albumin (Gibco, USA) to prevent nonspecific binding sites.

Subsequently, the tissue sections were incubated overnight at 4 C with primary antibodies. In the control group, an isotype control antibody solution was substituted for the primary antibodies. After rinsing, an anti-mouse fluorescein isothiocyanate (FITC)-conjugated secondary antibody solution was applied and allowed to incubate at RT for 45 min before removing unbound secondary antibodies with a PBS wash. To label nuclei, a solution of 4',6-diamidino-2-phenylindole was used. Fluorescent images were captured using a fluorescence microscope from the Optika Italy brand.

#### Spermatogonial survival

Promyelocytic leukemia zinc finger (PLZF) antibody at a dilution of 1/400 (orb100307, rabbit anti-PLZF antibody) served as an identifier for undifferentiated spermatogonia. Afterward, sections were treated with a secondary anti-mouse FITC-conjugated antibody (orb688925), followed by a 60-min incubation at RT. The findings were presented in terms of the average count of PLZF-positive cells per tubule.

#### Hypoxia index

Hypoxia-inducible factor 1-alpha (HIF-1-α), (rabbit anti-HIF-1-alpha antibody, 1/200, orb67372), was used as a marker of local-tissue oxygen tension. A secondary antibody labeled with FITC for mouse antigens (orb688925) was applied to the tissue sections and allowed to incubate for 60 min at RT. The findings were presented as the average count of HIF-1-α positive cells per tubule.

#### Sertoli cell functionality

Androgen-binding protein (ABP) (rabbit anti-ABP antibody, abin7072890) is synthesized by the Sertoli cell in the testis and is a marker for analysis of Sertoli cell function. A secondary anti-mouse FITC-conjugated secondary antibody (orb688925) was added to the sections and incubated for 60 min at RT. Results were expressed as the mean number of ABP-positive cells per tubule.

#### Terminal deoxynucleotidyl transferase dUTP nick end labeling (TUNEL) detection of apoptosis in grafted testicular tissue fragments

Paraformaldehyde-fixed and paraffin-embedded tissue sections underwent deparaffinization, followed by staining of apoptotic cells using the dead-end fluorescent TUNEL kit (G3250) (Promega Corporation, USA) according to the manufacturer's guidelines. Propidium iodide was used for total nuclei staining. Imaging was performed with a Zeiss Plan-Neofluar lens on an epifluorescence microscope at x20 magnification. A percentage was calculated by quantifying the ratio of TUNEL-positive nuclei to propidium iodide-positive total nuclei from 3 random fields for each treatment condition.

### Ethical Considerations

All experimental procedures and protocols were approved by the Animal Ethics Committee of Tehran University Medical Science, Tehran, Iran (Code: IR.TUMS.AEC.1401.001) with the guide for the care and use of laboratory animals published by the United States National Institutes of Health (NIH Publication, 8 ${ߐ}^{\text{th}}$ Ed, 2011).

### Statistical Analysis

Data were numerical and expressed as mean ± standard deviation. The Shapiro-Wilk test was used for normality testing, and homogeneity of variances was assessed with the Brown-Forsythe and Bartlett's tests. An ordinary one-way ANOVA was used to compare means among 5 treatment groups, with a p < 0.05 considered statistically significant. Statistical analyses were performed using GraphPad Prism (version X, GraphPad Software, LLC, San Diego, CA, USA).

## 3. Results

### Evaluation of decellularization protocol

**Figure 1:**
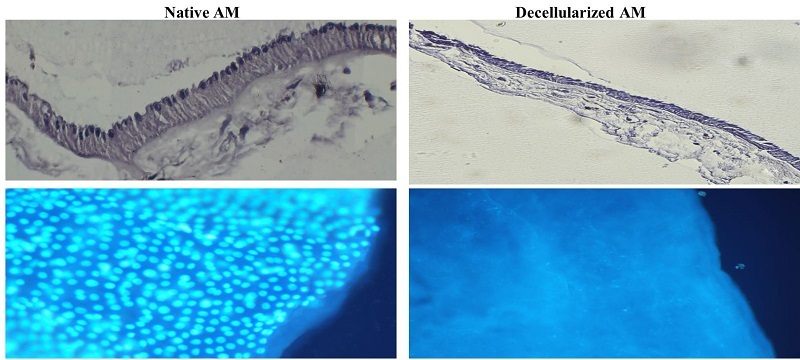
Visualization of decellularized AM using light and fluorescent microscope. (Top row) Hematoxylin and Eosin (H&E) staining of intact AM shows the presence of cellular components, while decellularized AM exhibits an absence of nuclei, confirming effective decellularization. (Bottom row) Hoechst nuclear staining further illustrates this, with intact AM showing prominent blue nuclear staining, whereas decellularized AM demonstrates no detectable nuclear signals, providing additional evidence for the removal of cellular material during the decellularization process. x20 magnification.

Histological analysis using H&E staining confirmed the effectiveness of the decellularization process. All nuclei were absent in the decellularized AM, indicating complete cellular removal. DNA staining further corroborated this finding, validating the absence of residual cellular material (Figure 1).

### Evaluation of hydrogels

Decellularized AM fragments were first digested to generate a solubilized ECM solution. Following pH and salt adjustments, 1 ml aliquots of this pre-gel solution were dispensed into micro tubes and incubated for 1 hr at 37 C. Gelation was confirmed by the solutions remaining static when the incubation plate was tilted, indicating that the ECM components had successfully formed a hydrogel network. Additionally, our previous studies have demonstrated that ECM components such as collagen, elastin, and glycosaminoglycans are preserved in AM hydrogel (18, 19).

### Seminiferous tubule integrity

**Figure 2:**
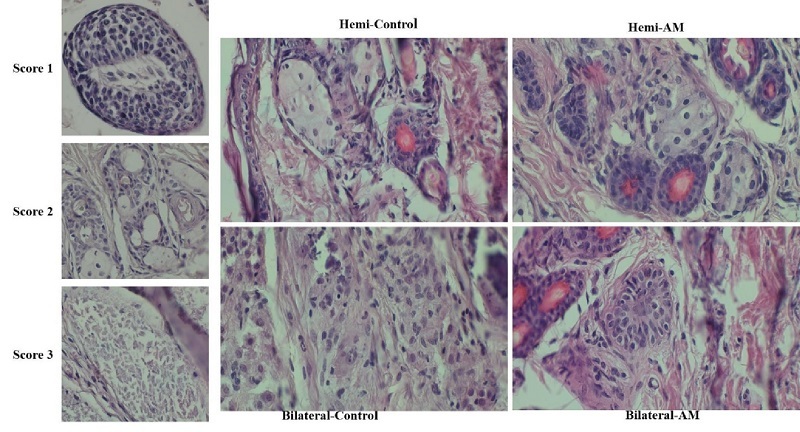
Impact of AM hydrogel encapsulation on seminiferous tubule integrity 21 days after grafting. The integrity of seminiferous tubules (STs) was evaluated 21 days post-grafting using H&E staining, and categorized based on their structural condition. Intact tubules (score 1) were characterized by cells adhering to the basement membrane, maintaining cohesive cellular architecture, and showing no signs of sclerosis. Satisfactory tubules (score 2) exhibited individualized intratubular cells despite the presence of focal necrosis. In contrast, damaged tubules (score 3) displayed complete necrosis, with no identifiable cellular structure or adherence to the basement membrane. This analysis demonstrates the impact of AM hydrogel encapsulation on preserving the structural integrity of (STs) during the grafting process. AM: Amnion membrane, Hemi: Hemicastration, Bilateral: Bilateral castration.

This study aimed to assess the effect of embedding testicular tissue fragments in an AM hydrogel on ST integrity. H&E staining of grafts harvested after 21 days post-transplantation was used to evaluate ST morphology. On day 21, the majority of STs exhibited satisfactory morphology (score 2) based on the scoring system. No significant difference was observed between nonencapsulated and AM hydrogel-encapsulated grafts when considering intact (score 1) or satisfactory (score 2) STs (Figure 2 and Table I) (p ≤ 0.05).

### Spermatogonial survival

**Figure 3:**
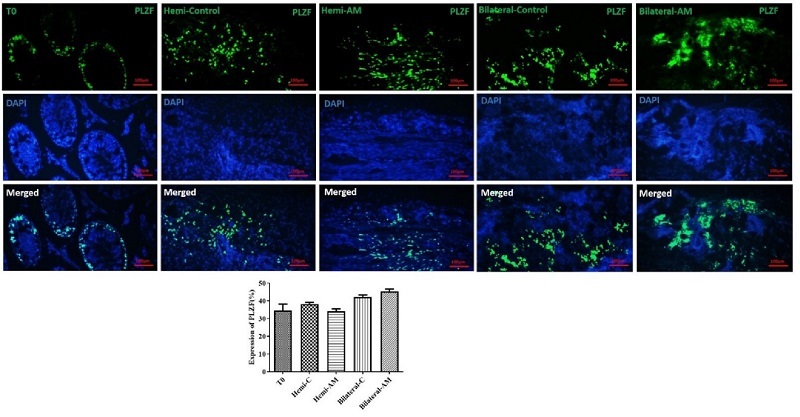
Detection of undifferentiated spermatogonia using immunostaining for PLZF. Immunostaining was performed to detect PLZF, a marker of undifferentiated spermatogonia. The analysis quantified the average number of PLZF-positive cells per ST, providing insights into the population of undifferentiated spermatogonia within the tubules. This evaluation highlights the preservation or changes in spermatogonial populations under the experimental conditions. Scale bars; 100 µm. AM: Amnion membrane, Hemi: Hemicastration, Bilateral: Bilateral castration, Hemi-C: Hemicastration-control and T0, tissue in day 0 (intact).

We examined a particular group of undifferentiated spermatogonia, which includes SSCs. Immunohistochemistry for PLZF was utilized to measure their presence in the grafts (Figure 3). The number of PLZF-positive cells per ST after 21 days post-transplantation did not exhibit a significant difference between nonencapsulated and AM hydrogel-encapsulated groups (p ≤ 0.05).

### Sertoli cell functionality

**Figure 4:**
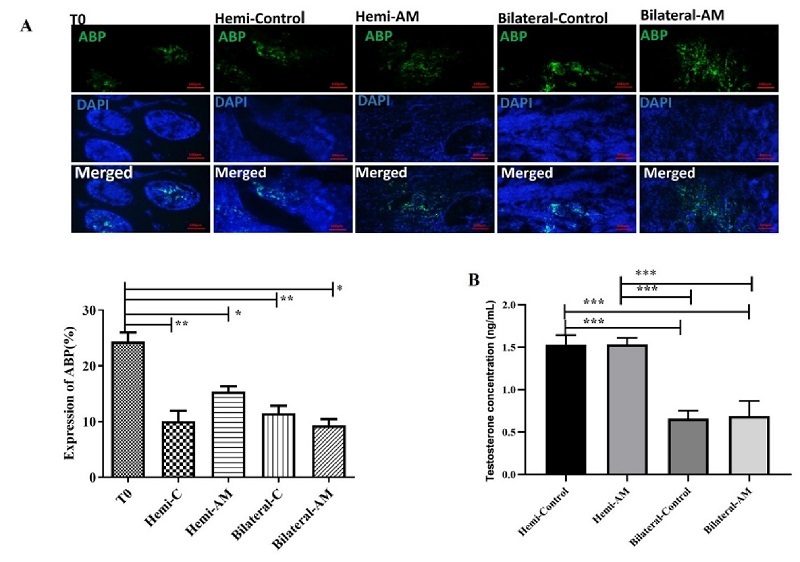
A) Mouse testicular tissue fragments were encapsulated in AM hydrogel or left unencapsulated. After 21 days of transplantation, the harvested grafts were stained for the ABP marker to assess Sertoli cell functionality. B) Additionally, serum testosterone concentration was evaluated in the different groups at the end of day 21. Scale bars; 100 µm. *P ≤ 0.05, **P = 0.001, ***P = 0.0001. AM: Amnion membrane, Hemi: Hemicastration, Bilateral: Bilateral castration, Hemi-C: Hemicastration-control and T0, tissue in day 0 (intact).

The Sertoli cells generate a protein known as ABP that attaches to testosterone, ensuring a high testosterone level is maintained within the testes. This process is crucial for supporting spermatogenesis. Therefore, the evaluation of ABP by immunohistochemistry assay is a suitable marker for Sertoli cell counting and functionality in the harvested testis tissues. We observed that the number of Sertoli cells in all of the groups than nongrafted testicular tissues on day zero (T0) significantly decreased (p < 0.001) (Figure 4A). In addition, the results of testosterone concentration in the serum of treated mice showed that a significant difference was observed in mice under bilateral or hemi castration (p < 0.0001) (Figure 4B).

### HIF-1α and immunohistochemistry assay

**Figure 5:**
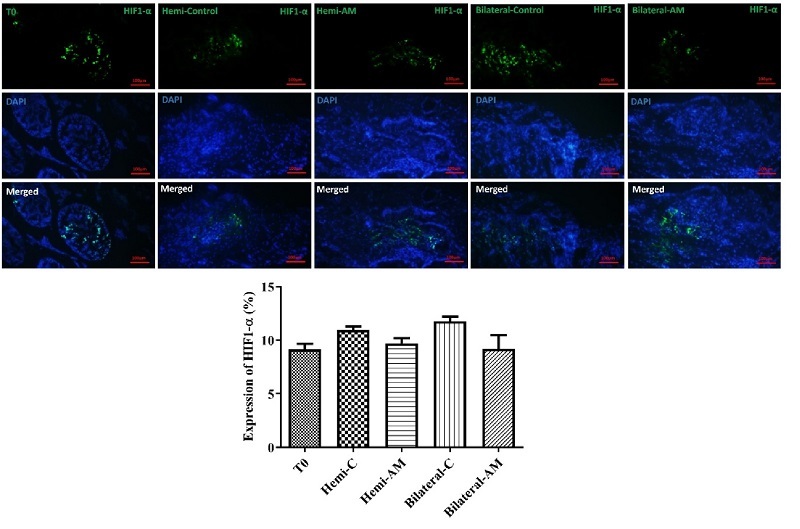
Assessment of hypoxia in grafted testicular tissues using HIF-1α immunohistochemistry. A hypoxia assay was conducted to evaluate the levels of hypoxia in testicular tissues grafted with or without AM hydrogel encapsulation. Immunohistochemical staining for HIF-1α was performed to detect hypoxic regions in the grafted tissues. The analysis included tissues grafted into hemi-castrated and bilaterally castrated mice, allowing for a comparative evaluation of hypoxia between encapsulated and noncapsulated grafts under different experimental conditions. Scale bars; 100 µm. AM: Amnion membrane, Hemi: Hemicastration, Bilateral: Bilateral castration, Hemi-C: Hemicastration-control and T0, tissue in day 0 (intact).

HIF-1α and HIF-2α subunits form a heterodimeric transcription factor that regulates cellular responses to hypoxia. We measured HIF-1α expression in grafted testicular tissue, finding no significant difference between unencapsulated grafts and those encapsulated with AM hydrogel (p ≤ 0.05) (Figure 5).

### Apoptosis assay

**Figure 6:**
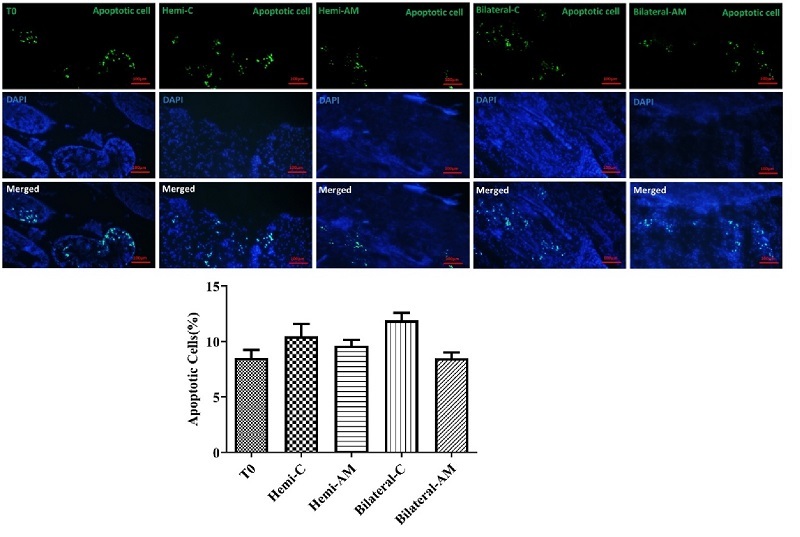
Detection of apoptotic cells in autografted testicular tissues using TUNEL assay. Apoptotic cells were identified in testicular tissues autografted into hemi-castrated and bilaterally castrated mice under 2 experimental conditions: AM hydrogel encapsulation and noncapsulated groups. The TUNEL assay was used to detect DNA fragmentation, a hallmark of apoptosis, providing insights into cell survival and the impact of AM hydrogel encapsulation on the rate of apoptosis in grafted testicular tissues. Scale bars; 100 µm. AM: Amnion membrane, Hemi: Hemicastration, Bilateral: Bilateral castration, Hemi-C: Hemicastration-control and T0, tissue in day 0 (intact).

On day 21, undifferentiated apoptotic cells were detected with a TUNEL kit in all grafted groups. The mean number of apoptotic cells per tubule was similar in nonencapsulated and AM in both mice undergoing hemi or bilateral castration (p ≤ 0.05) (Figure 6).

**Table 1:** Impact of AM hydrogel encapsulation on ST integrity 21 days after grafting

**Condition**	**Hemi-control**	**Hemi-AM**	**Bilateral-control**	**Bilateral-AM**	**P-value**
**Score 1 (Intact) (%)**	0.95 ± 0.71	0.85 ± 0.59	0.80 ± 0.44	0.93 ± 0.40	< 0.001
**Score 2 (Satisfactory) (%)**	56.51 * ± * 10.93	59.51 * ± * 13.65	53.46 * ± * 12.80	54.51 * ± * 15.65	≤ 0.05
**Score 3 (Damaged) (%)**	48.49 * ± * 15.93	39.45 * ± * 12.93	45.51 * ± * 10.93	44.44 * ± * 14.93	≤ 0.05
Data presented as mean ± SD, one-way ANOVA test. ST: Seminiferous tubule, AM: Amnion membrane, Hemi: Hemicastration, Bilateral: Bilateral castration					

## 4. Discussion

This study investigates the hypothesis that hAM hydrogel, rich in diverse growth factors including pro-angiogenic factors like platelet-derived growth factor, vascular endothelial growth factor, transforming growth factor beta, and possessing anti-inflammatory properties, can mitigate some of the complications associated with ischemia (17). In addition, having different types of ECM proteins that contain cell adhesion motifs can accelerate the migration and recruitment of endothelial cells, fibroblasts, and vascular smooth muscle cells to the grafted tissue (20). However, the results revealed no difference in the survival rate of spermatogonial cells, ischemia rate, and apoptosis between the encapsulated group in AM gel and the nonencapsulated group.

Our findings and a literature review suggest that strategies focused solely on promoting neovascularization to mitigate hypoxia, especially during the early stages of transplantation, may have limited effectiveness. The process of angiogenesis and anastomosis of the small vessels of the grafted tissue with the large vessels of the host is time-consuming, requiring migration and proliferation of host endothelial cells into the grafted tissue, followed by their spatial organization to form small vascular-like structures (21). Additionally, the newly formed vasculature after transplantation is fragile, lacking the strength to resist the elastic nature of the skin tissue or the pressure exerted by the recipient's mobility (5).

Several studies have explored the use of hydrogels, including alginate loaded with nanoparticles carrying angiogenic factors or apoptosis inhibitors, and matrigel hydrogel to enhance angiogenesis (13, 22). While these treatments demonstrated increased vascular density in transplanted tissues over longer durations (day 21), they did not significantly improve outcomes within the first day post-transplantation. Therefore, these approaches fail to alleviate the effects of acute hypoxia during this critical period. While these interventions demonstrate increased vascular density over longer durations (day 21), they fail to alleviate acute hypoxia during the initial post-transplantation period.

Notably, most studies used tissue fragments of 1–3 mm^3^, a size that allows for passive diffusion of gases, electrolytes, and nutrients. Even in a study using large pieces of immature macaque testicular tissue transplanted under the dorsal skin, acute hypoxia and the lack of a vascular network between the grafted tissue and the host did not impede spermatogenesis and sperm production after 6 months (6). These findings suggest that factors beyond hypoxia induced by avascular transplantation contribute to reduced spermatogonial survival and ST integrity. Changes in the biomechanical environment of the grafted STs likely play a significant role in the fate of the transplanted tissue. Within the testis, the STs are encased by a robust fibrous membrane, the tunica albuginea, a thick and collagenous matrix (23).

The tunica albuginea, a tough fibrous capsule surrounding the testis, plays a crucial role in testicular function. It exerts the necessary mechanical force required for the formation and maturation of testicular lobules (24). The forceful expulsion of STs observed upon puncturing the albuginea with a sharp object further supports the notion of substantial internal pressure and biophysical forces within the testis. These forces are likely attributable to the structural properties of the tunica albuginea. Conversely, mechanical forces and stimuli are transduced into intracellular biochemical signals by mechanoreceptors, specialized cellular structures that ultimately influence cell behaviour and function (25). Notably, proteins involved in cell-cell adhesion (adherens junctions) and intercellular communication (gap junctions) function as mechanoreceptors (26).

These proteins play a critical role in connecting Sertoli cells and germ cells at various stages of development. Furthermore, they are essential for compartmentalizing and polarizing the cytoplasm within Sertoli cells, thereby establishing the appropriate microenvironments necessary for germ cell development (27). These findings suggest that mechanoreceptors on testicular cells are sensitive to changes in the biomechanical environment, potentially impacting cellular function. A case study highlighting this concept is a 45-yr-old man with azoospermia and spermatogenic arrest who lacked a tunica albuginea in his right testicle (28). The removal of the albuginea during subcutaneous transplantation likely alters the mechanical properties experienced by the STs.

Given that the testis and skin have distinct mechanical characteristics, this suggests that biomaterials used for testicular xenograft or autograft encapsulation should be designed to replicate, to a significant degree, the mechanical environment of the native testis. The study investigating hydrogels for testicular tissue autotransplantation have shown that alginate hydrogels outperform fibrin matrices, even when incorporating nanoparticles containing angiogenic factors. While fibrin offers superior biological properties, alginate exhibits significantly different mechanical characteristics. Notably, the 1% alginate used in this study was stiffer and rougher than the fibrin matrix, as evidenced by its higher G ' modulus (4500 Pa vs. 900 Pa) (22). Furthermore, studies employing Matrigel around autologously grafted testicular tissues in Rhesus macaques observed no improvement in the percentage of tubules with complete spermatogenesis (6). These findings collectively highlight the importance of evaluating biomaterials with diverse biomechanical properties in transplantation studies. The ideal biomaterial should closely resemble the mechanical environment and biophysical forces experienced within the native testis. This study was originally planned to focus on the biological properties of hydrogels derived from acellular tissues. However, its results and previous studies suggest a hypothesis that the mechanical structure of these hydrogels, including stiffness, roughness, and stability, may play a more significant role in maintaining the spatial structure of STs. Therefore, future research should consider the limitations of this study, particularly by examining the mechanical properties of hydrogels.

This could involve comparing protein-based hydrogels with synthetic and natural hydrogels that have a polysaccharide base structure or using cross-linkers like 1-ethyl-3-(3-dimethylaminopropyl) carbodiimide-N-hydroxysuccinimide, as well as Genipin, to enhance the mechanical properties of protein-based hydrogels in approaches for testis transplantation (29). Investigating the effect of biomaterials with different mechanical properties on the function, phenotype, and behavior of Sertoli cells -which contain the most mechanoreceptor proteins- can provide a clearer understanding of how physical forces maintain and influence the special arrangement of testicular cells in STs. In conclusion, previous studies have employed various hydrogels for testicular tissue transplantation, including protein-based hydrogels (e.g., Matrigel) and polysaccharide-based hydrogels (e.g., alginate). Notably, these studies showed that polysaccharide-based hydrogels yield superior outcomes in transplanted tissue. This effectiveness can likely be attributed to their favorable mechanical properties despite their reduced biological activity compared to protein-based hydrogels.

## 5. Conclusion

This study explores the use of acellular tissue-derived hydrogel for testicular tissue transplantation but found no significant improvement in spermatogonial survival, ischemia, or apoptosis between encapsulated and nonencapsulated groups. The results suggest that the hydrogel's biological properties may have a limited impact on addressing acute hypoxia during the early stages. Additionally, the study highlights the importance of the mechanical properties of hydrogels, such as stiffness and roughness, in maintaining ST integrity. Future research should focus on enhancing these mechanical characteristics to better mimic the native testicular environment.

##  Data Availability

Data will be made available on request via email to corresponding author.

##  Author Contributions

K. Gholami: Conceptualization, writing original draft, methodology; E. Asheghmadine, F. Guitynavard, L. Zareian Baghdadabad, D. Taheri, and P. Zahmatkesh: Methodology, date collection, date analysis, review the paper; LO. Reis: Validation, experiment design, date analysis; SMK. Aghamir: Project management, validation, final review.

##  Conflict of Interest

The authors declare that there is no conflict of interest.
